# SARS-CoV-2 Immunization Index in the Academic Community: A Retrospective Post-Vaccination Study

**DOI:** 10.3390/idr16060088

**Published:** 2024-11-25

**Authors:** Keltyn Oliveira, Ana Almeida, Carina Silva, Miguel Brito, Edna Ribeiro

**Affiliations:** 1 Health & Technology Research Center, Escola Superior de Tecnologia da Saúde, Instituto Politécnico de Lisboa, Av. D. João II, Lote 4.69.01, Parque das Nações, 1990-096 Lisboa, Portugal; ketlyn.oliveira@ctn.tecnico.ulisboa.pt (K.O.); carina.silva@estesl.ipl.pt (C.S.); miguel.brito@estesl.ipl.pt (M.B.); 2 Escola Superior de Tecnologia da Saúde, Instituto Politécnico de Lisboa, Av. D. João II, Lote 4.69.01, Parque das Nações, 1990-096 Lisboa, Portugal; ana.almeida@estesl.ipl.pt; 3 Centro de Estatística e Aplicações, Faculdade de Ciências, Universidade de Lisboa, 1749-016 Lisboa, Portugal

**Keywords:** SARS-CoV-2, immunity, vaccines, academic community, immunological variables

## Abstract

Background/Objectives: The COVID-19 pandemic has revolutionized vaccine production and compelled a massive global vaccination campaign. This study aimed to estimate the positivity and levels of SARS-CoV-2 IgG antibodies acquired due to vaccination and infection in the academic population of a Portuguese university. Methods: Blood samples were collected and analyzed through the ELISA methodology, and statistical analysis was performed. Results: A total of 529 volunteers with at least one dose of the vaccine were enrolled in this study. Individuals without a prior COVID-19 diagnosis were divided into two groups: 350, who received a full vaccination, and 114, who received a full vaccination and a booster dose of the same vaccine (81) and mixed vaccines (33). Regarding the individuals who reported a prior SARS-CoV-2 infection, 31 received a full vaccination, and 34 received only one vaccination dose. Data analysis showed a higher level of IgG against SARS-CoV-2 in individuals who were younger, female, who received the Moderna vaccine, with recent post-vaccine administration, a mixed booster dose, and prior SARS-CoV-2 infection. Conclusions: Assessing vaccination’s effectiveness and group immunity is crucial for pandemic management, particularly in academic environments with high individual mobility, in order to define groups at risk and redirect infection control strategies.

## 1. Introduction

In December 2019, several instances of severe pneumonia with a viral origin in the Chinese city of Wuhan were reported [1]. Due to its high similarity to the severe acute respiratory coronavirus (SARS-CoV), which caused outbreaks between 2002 and 2004 [2], the novel viral agent identified was designated SARS-CoV-2.

The emerging virus belongs to the family Coronaviridae, and, similar to other coronaviruses, it is an enveloped positive-sense single-stranded RNA virus [3]. SARS-CoV-2 encodes a wide range of proteins, including the Spike glycoprotein, which is the main factor in coronavirus tropism [4]. The Spike protein was identified as an antigenic target for the development of vaccines against SARS-CoV-2 since the antibodies directed to this protein have been shown to neutralize the virus [5,6,7,8].

COVID-19 (coronavirus infectious disease 2019) caused by the SARS-CoV-2 virus was acknowledged as an outbreak of public health emergency of international concern (PHEIC) by the World Health Organization (WHO) on 30 January 2020, which quickly spread worldwide, causing a pandemic declared on 11 March 2020, that reached approximately 545 million confirmed cases and 6.33 million deaths [9,10,11]. SARS-CoV-2 arrived in Portugal on 2 March 2020, when the first two cases were confirmed [12]. In two years of the pandemic, Portugal had roughly 4.89 million confirmed COVID-19 cases and 23,490 deaths [9]. Overall, only 14% of individuals infected with SARS-CoV-2 develop severe disease with a significant need for health care assistance, including intensive hospital treatment. However, the associated high transmission rates of SARS-CoV-2 led to a high number of patients seeking hospital care, which represented a massive burden for national health systems, many of which have nearly collapsed in countries that have been severely affected by the pandemic [13].

The accelerated worldwide spread of the virus and the continuing pandemic are mediated, in part, by the absence of SARS-CoV-2 pre-existing immunity since the presence of antibodies acquired after infection or vaccination that affects the transmission of the virus, the severity of illness, and the probability of clinical reinfection [14,15,16,17].

With the reopening of schools and universities after the COVID-19 pandemic, research aiming to understand the transmission dynamics in this particular academic population was performed. Although a few studies have shown no association between school in-person attendance and the increase in transmission of the SARS-CoV-2 infection [18,19], other studies reported large outbreaks associated with the reopening of schools [20]. Moreover, higher infection rates are closely related to the efficiency of viral transmission of SARS-CoV-2 from symptomatic and asymptomatic individuals in crowded and limited indoor spaces or indoor activities [21], including classes. To guarantee the safe reopening of educational institutions, new research aiming at SARS-CoV-2 seroprevalence among students, professors, and staff members was essential.

Since December 2020, the European Medicines Agency’s scientific recommendations led to the approval of six different COVID-19 vaccines [22]. The Portuguese vaccination program began in January 2021 and, by June 2021, has delivered over 9.72 million first doses, standing out internationally as one of the countries with the highest vaccination rate [23].

Approximately 87.28% of the Portuguese population completed the initial COVID-19 vaccination protocol [23], predominantly using the first four vaccines approved in Europe: BNT162b2 (Pfizer/BioNTech), ChAdOx1 (AstraZeneca, AZ), mRNA-1273 (Moderna) and Ad26.COV2.S (Johnson & Johnson/Janssen). BNT162b2 and mRNA-1273 are mRNA vaccines that use lipid nanoparticles to deliver mRNA that encodes for a pre-fusion stabilized SARS-CoV-2 Spike protein [24,25]. In contrast, Ad26.COV2.S and ChAdOx1 are non-replicating adenoviral vector vaccines based on an incompetent adenovirus that encodes a wild-type version of the SARS-CoV-2 Spike protein [26,27]. Despite the divergence of the approved and utilized vaccines, namely, mRNA vaccines and non-replicating adenoviral vector vaccines, and the fact that vaccination schemes may include crossed vaccination (mRNA and viral vaccines), there are no doubts regarding its value for the global control of COVID-19 [28,29], and although the duration of immunity protection is still unclear, studies suggest that it may last for at least 5–6 months [30,31].

Several studies that focused on post-immunization antibody titers dada analysis have described and established the COVID-19 vaccine’s protective effectiveness, and the use of these studies was supported as the basis for the development of a consensual correlate of protection (CoP) [32,33]. Additionally, evidence that antibody levels wane over time [34,35,36,37,38] highlights the importance of surveillance to define appropriate immunization strategies since the future dynamics of SARS-CoV-2 circulation may be predicted by the expected length of SARS-CoV-2 immunity. Moreover, due to differences in formulation, mRNA dose, or timing of the prime-boost regimen, even vaccines with the same mechanism of action may differ in their ability to provide clinical protection [39].

Here, we used a semi-quantitative binding assay to retrospectively measure IgG against SARS-CoV-2 Spike protein to evaluate the magnitude of antibody responses in the academic community of Instituto Politécnico de Lisboa (IPL). The type of vaccine, time after vaccination, age, and sex were explored as variables that could impact the antibody response. Moreover, antibody levels following vaccination were compared to the IgG response generated in people who have been infected with SARS-CoV-2. This study focused on the assessment of IgG antibody prevalence against SARS-CoV-2 after vaccination in the IPL academic population.

## 2. Materials and Methods

### 2.1. Participants Enrolled in This Study

This study was carried out between December 2021 and February 2022 in IPL, located in Lisbon, Portugal. Among 1050 workers, 529 volunteers (50.4%) aged 17 to 76 years old, including students, professors, and school staff from IPL, were enrolled in this study. Workers of all units of the engaged Public Higher Education Institution from Portugal were invited to voluntarily participate in the surveillance project. Before enrollment in the screening tests, all volunteers filled out a written informed consent and a questionnaire. This survey instrument consisted of a questionnaire assessing contact information, sociodemographic characteristics (age, sex, nationality, ethnicity, household size), vaccination (including the number of shots, dates of administration, and type of commercial vaccine), and COVID-19 exposure characteristics (previous diagnostic, symptoms, and history of close contact with COVID-19 cases). A complete vaccination scheme, according to Portuguese health authorities and vaccine administration guidelines at the time, consisted of two doses of the commercial vaccines from Pfizer, Moderna, and AstraZeneca and a single dose of the Janssen vaccine. Individuals were considered fully vaccinated at least 14 days after the vaccine administration. Regarding the individuals who received a boost dose, since antibody production is known to be rapid, reaching a peak between 6 and 7 days, the boost dose was considered at least 5 days after the vaccine administration [40].

### 2.2. Antibody Detection

Venous blood was collected through venipuncture according to standard protocols in a dry tube (serum/plasma) for IgG quantification. Analytical tests were performed using Optic Ivyman System 2100C equipment, employing an Enzyme-Linked Immunosorbent Assay (ELISA) in plasma samples (K3 EDTA). IgG antibodies against the S1 subunit of the virus S protein were semi-quantified using a commercial EUROIMMUN Anti-SARS-CoV-2 ELISA (IgG) kit. The default result units for this SARS-CoV-2 IgG assay are the index calculated by sample optical density (OD) ratio over the calibrator OD. According to the manufacturer’s instructions, IgG results were deemed positive if the cut-off index (S/C) was ≥1.1, while negative results were defined by a cut-off of <0.8. Borderline values (index ≥ 0.8 to <1.1) were considered inconclusive results.

### 2.3. Statistical Methods

Data obtained from questionnaires were subsequently entered into an anonymous questionnaire programmed in REDCap (Research Electronic Data Capture) to estimate the prevalence of individuals with SARS-CoV-2 antibodies in the studied population. Statistical analysis was performed using SPSS^®^ Statistics version 26.0 (IBM, Armonk, NY, USA), and it was considered to be at a significance level of 0.05. Counts and proportions (n [%]) were reported for categorical variables, and quantitative data were described using mean and standard deviation (SD) values. Comparison of quantitative data among categories of the most relevant covariates was conducted through a one-way ANOVA analysis, two-way ANOVA, Kruskal–Wallis H test, and *t*-test for paired samples, whenever appropriate.

### 2.4. Ethics Statement

This work is included in a project from the IPL (COVIDVax-IPL), approved by the Ethics Committee of Escola Superior de Tecnologia da Saúde de Lisboa (ref: CE-ESTeSL-Nº. 91-2021). All volunteers provided a signed written informed consent before enrolment in this study, following the Helsinki Declaration and Oviedo Convention in agreement with the Portuguese General Data Protection Regulation (GDPR) law nº 58/2019 from 8th August regarding data protection.

## 3. Results

We performed a semi-quantitative binding assay to measure IgG against SARS-CoV-2 Spike protein to access the immunization index in the academic community of IPL. Such variables as type of vaccine, time after vaccination, age;], sex, and previous COVID-19 diagnosis were explored as variables with potential impact on the immune response. Data from 529 volunteers were retrospectively analyzed, with an average age of 38 (±15.3), and 68% were female. Data revealed that SARS-CoV-2 IgG antibody positivity ranged from 1.2 to 16.1. Questionnaire data analysis revealed that six individuals (1.1% of the study population) had positive antibody titters without receiving a vaccine, indicating that they had been infected with SARS-CoV-2 at some point before the time of collection. After the first, second, and third vaccinations, 34, 31, and 128 individuals had previous COVID diagnoses, respectively, and 1, 327, and 2, respectively, did not. The variables sex, age, and vaccine boost induced significant effects on immunization indexes.

### 3.1. Sample Characterization and Detected Immunization Index

The volunteers enrolled in this study were 359 females and 170 males. Data were stratified by vaccination program and COVID-19 prior infection (fully vaccinated; fully vaccinated and boosted (same vaccine); fully vaccinated and boosted (mixed vaccine); fully vaccinated with prior COVID-19 infection; with one dose and COVID-19 prior infection). It was also stratified by sex and age groups (establishing the following age ranges in years: 17–32 (n = 214); 33–47 (n = 141); 48–62 (n = 149); and 63–76 (n = 25)).

The SARS-CoV-2 IgG index levels are summarized in Table 1 by sample demographic characteristics, vaccination scheme, and diagnosed infection.

### 3.2. SARS-CoV-2 Immunization Index Comparison Analysis Between Sex and Age

**Figure 1:**
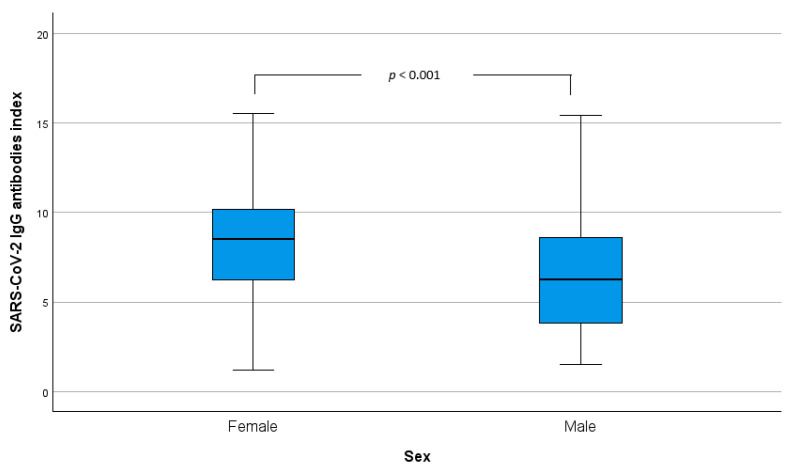
SARS-CoV-2 IgG antibodies index boxplots by sex for fully vaccinated individuals. The *p*-value obtained by *t*-test.

In fully vaccinated individuals, female (F) participants had an index average of SARS-CoV-2 IgG antibodies of 8.3 (±2.8), while male (M) participants had an index average of 6.5 (±3.2). Figure 1 depicts the boxplots of the index of SARS-CoV-2 IgG antibodies by sex, and the difference in IgG antibody mean levels between the groups was found statistically significant (*z* = 5.4, n_F_ = 248; n_M_ = 102, *p* < 0.001).

Regarding the different age groups, we obtained an index mean of 8.4 (±3.2) for individuals aged between 17 and 32, an index mean of 7.2 (±2.6) for individuals aged 33 to 47, and an index mean of 7.1 (±3.0) for individuals aged 48 to 62. Following the full vaccination, IgG antibody levels differed significantly between age groups.

**Figure 2:**
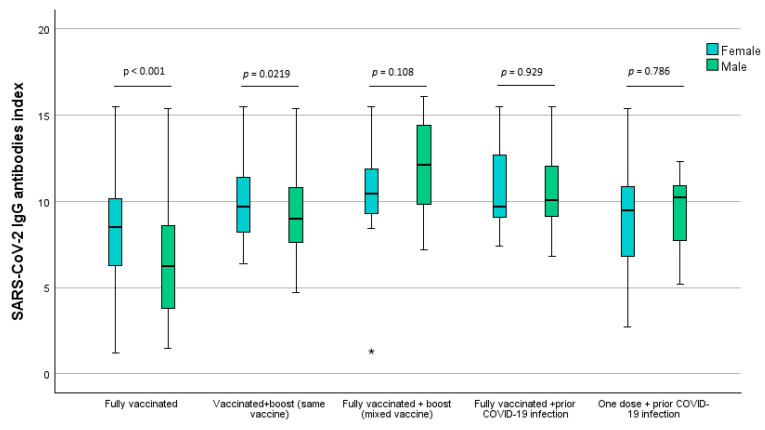
SARS-CoV-2 IgG antibodies index comparison between sex and vaccination scheme. *p*-values obtained using Sidak’s multiple-test adjustment. * Severe outlier.

Using a two-way ANOVA analysis, it was found that there was a significant interaction between the vaccine group’s program and sex (F = 3.77, *p* = 0.005). As we can observe in Figure 2, the index distribution among male and female participants is quite particular. Using Sidak’s multiple testing adjustment, it was found that our data demonstrated that the SARS-CoV-2 immunization index was significantly different for both males and females only in individuals with complete vaccination programs (*p* < 0.001), wherein females endorsed initially higher SARS-CoV-2 IgG antibodies index after a full vaccination program. Males were revealed to endorse higher immunization levels after a complete vaccination program and additional boost with mixed vaccines but with no significative differences (*p* = 0.108).

**Figure 3:**
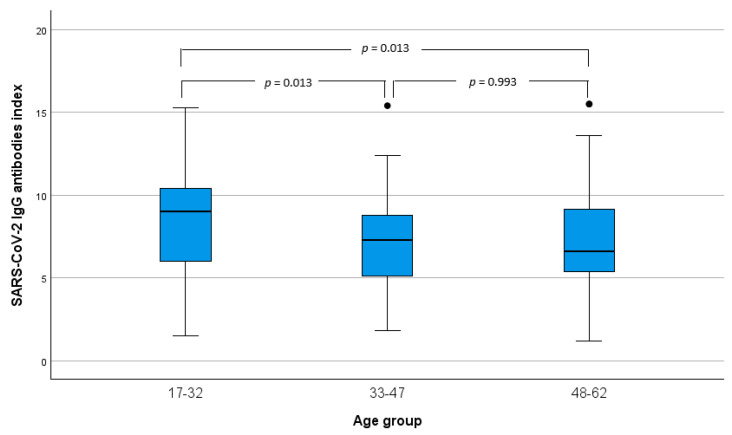
SARS-CoV-2 IgG antibodies index boxplots by age group. *p*-values obtained by Dunnett’s multiple testing adjustments. ● moderate outliers.

Moreover, considering the participants who were fully vaccinated, we compared the SARS-CoV-2 IgG antibodies index mean production in the different age groups. Figure 3 depicts the boxplots of SARS-CoV-2 IgG antibodies index by age group, and using a one-way ANOVA analysis, it was found that there were significant differences between the mean values (F = 6.86, *p* = 0.001). Using Dunnett’s multiple testing adjustment, it was found that the significant differences were between 17–32 and 33–47 and between 17–32 and 48–62 age groups. In this vaccination scheme group, there were no participants in the class of 63–76 years.

**Figure 4:**
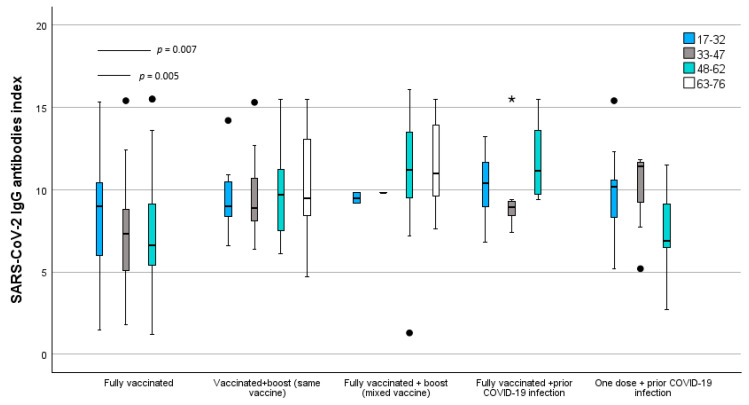
SARS-CoV-2 IgG antibodies index comparison between age groups and vaccination scheme. *p*-values obtained using Sidak’s multiple-test adjustment. ● moderate outliers * severe outliers.

Interestingly, SARS-CoV-2 immunization response distribution is also divergent between age groups, as we can observe in Figure 4, with similar patterns of response for 17–32 and 33–47 age groups and a very distinct pattern for participants older than 48 years old. This divergence is particularly noticeable in individuals with a complete vaccination program before COVID-19 infection, with associated higher immunization indexes for participants older than 48 years than younger individuals and conversely lower SARS-CoV-2 IgG antibodies levels with only one vaccine dosage before COVID-19 infection. Using Sidak’s multiple testing adjustment, statistical differences were found only between the groups aged 17–32 and 33–47 (*p* = 0.005) and 17–32 and 48–62 (*p* = 0.007) in the vaccine group of fully vaccinated individuals. It was not possible to apply the tests to the group of Fully vaccinated + prior COVID-19 because the sample sizes were too low.

### 3.3. SARS-CoV-2 Immunization Index Comparison Analysis Between Scheme Vaccination Groups

**Figure 5:**
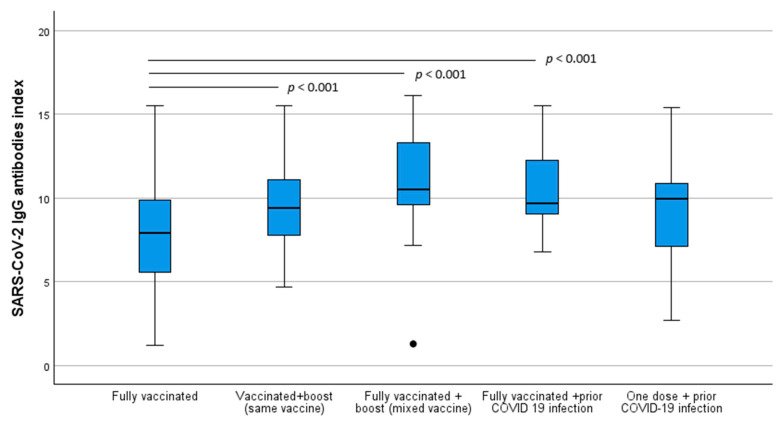
SARS-CoV-2 IgG antibodies index boxplots by scheme vaccination. *p*-values were obtained through Bonferroni’s adjustments. ● is a severe outlier.

Here, we observed that the SARS-CoV-2 immunization index varied significantly in individuals fully vaccinated in comparison with individuals with boost vaccination, independently of the type of boost vaccine and with prior COVID-19 diagnosis. Interestingly, no significant differences were observed in the SARS-CoV-2 immunization index of fully vaccinated individuals when compared with participants who had one vaccination dose and prior COVID-19 infection. On the other hand, regarding the other analyzed groups, a statistically significant difference was found in the mean values of the index of the SARS-CoV-2 IgG antibodies between the groups (F = 20.7, *p* < 0.001). Through Bonferroni’s adjustments analysis, significant differences were found (data are summarized in Figure 5).

Regarding the 114 participants that had a boost vaccine, the mean levels of the SARS-CoV-2 IgG antibody index obtained when the same vaccine type of the prior vaccination program was administered were 9.9 (±2.7), while individuals that had the boost dose made with a different type of vaccine had an index mean value of 11.1 (±3.1).

Furthermore, the difference between the mean values of the IgG antibodies index produced in participants who had prior COVID-19 infection and were fully vaccinated compared to the index in individuals who had prior COVID-19 infection but received only one vaccine dose was statistically significant (*z* = 2.4, n_1_ = 31, n_2_ = 34, *p* = 0.020).

## 4. Discussion

The emergence of the COVID-19 pandemic has revolutionized the time it took for vaccines to become available to the global population, and there has been an unprecedented urgency for vaccines to be administered to prevent the mortality caused by the virus [41]. However, it is vital to understand vaccination’s effectiveness and potential causes in individual variability responses. Despite the number of studies conducted to date, including ours, it is impossible to infer a causal relationship between the levels of antibodies measured and the efficacy of the vaccines or the protection offered from infection with SARS-CoV-2 [42]. Concurring with this difficulty is that although SARS-CoV-2 has undergone numerous mutations over the last few years, the truth is that the vaccines administered have remained similar since the beginning of the worldwide vaccination process [43]. As such, we cannot say with certainty that the value of antibodies in each individual is efficient for most of the new variants, nor do we know if the number of antibodies directly influences the defense against the infection [42].

Considering that pre-immunization SARS-CoV-2 IgG antibody levels quantified in academic environments were relatively low [44] and that the higher percentages of seropositive students were reported at the end of the semester [45], in order to ensure the safe reopening of educational institutions, research aiming at SARS-CoV-2 seroprevalence among students, professors, and staff members was crucial. Here, we performed a semi-quantitative binding assay to retrospectively measure IgG against SARS-CoV-2 Spike protein to evaluate the magnitude of antibody responses in the academic community of IPL resultant from different vaccination schemes available at the time of sample collection. Analyzed data from IPL volunteers enrolled in this study were used to assess the prevention of the spread of infection in academic populations and allowed us to infer the prevalence of SARS-CoV-2 immunity within the IPL academic community.

In order to provide data for the evaluation of the general immune response in an academic population from IPL in Lisbon, SARS-CoV-2 vaccines were retrospectively analyzed, and variables, including type of vaccine, time after vaccination, age, sex, and previous COVID-19 diagnosis, were explored as variables with probable impact in the immune response.

Since all 529 participants in this research received at least one dose of the COVID-19 vaccine, an immunological response was expected. All plasma samples analyzed by ELISA were found to be positive, and SARS-CoV-2 IgG antibody levels ranged from 1.2 ± 3.2 to 16.1 ± 3.2. The results revealed that vaccination could induce strong humoral immune responses and that the levels of IgG antibodies were influenced by several factors, including age, gender, type of vaccine, and prior infection.

Analyzing the levels of IgG antibodies between fully vaccinated individuals with no prior COVID-19 diagnostic, we found a significant difference in the capacity for antibody production according to gender. The ability to produce antibodies was found to be higher in females than in males.

A similar disparity between the genders was discovered earlier for both Pfizer and AstraZeneca vaccinations, indicating that women were more likely than men to have high levels of antibodies against SARS-CoV-2 [46,47]. Previous research has shown that females had stronger protective antibody responses than males in response to a variety of vaccinations [48,49]. Although the exact processes are unknown, genetic and hormonal factors are suggested as possible explanations [50].

Moreover, a significant difference in antibody production between age groups was found, indicating that age could affect the levels of antibodies produced. Analyzing our data, we can observe a significant decrease in antibody levels according to ascending age groups.

In fact, the gradient of age response following vaccination, where older people have a lower capacity for antibody production, has been seen in several studies regarding SARS-CoV-2 vaccines [51,52,53]. The vast majority of studies related to antibody levels suggest that the immune response decreases with age due to the loss of antibody-producing cells over time [51,54,55].

The influence of age is also highlighted when we restricted our analyses to people who were 1–4 months post-full vaccination and compared them to the group who had the booster dose within the same month period, and we found a mean index of 10.4 ± 1.7 in the first group and a mean index of 9.9 ± 2.7 in the second group, with no significant difference between the values. The group of individuals fully vaccinated with a booster dose was mostly composed of older individuals; 53 of the 81 individuals were older than 48, and 15 of them were older than 63. In contrast, in the group without the booster dose, 42 of the 45 individuals were aged between 17 and 32, and no individual was older than 63.

Considering the main difference between these two groups, despite taking a booster vaccine, this could indicate the importance of booster vaccines for older individuals, as they required a booster vaccine to achieve the same levels of antibodies as younger people with a full vaccination scheme.

Other studies found that antibody levels after an infection or vaccine administration decreased over time [56], especially among older individuals and males [57,58,59]. The higher immunological response of Moderna compared to other vaccines has been reported in several studies [60,61,62]. Additionally, although Janssen vaccines are effective against symptomatic and asymptomatic SARS-CoV-2 infection [27], several studies reported a weaker immune response compared to other vaccines [63,64]. Previous research has shown that heterologous COVID-19 vaccines significantly increased IgG antibodies, neutralizing antibodies, and the cellular immune response compared to the homologous strategy [65,66,67]. When we compared the group of individuals who had received a booster dose of the same commercial vaccine to a group of individuals who had received a booster dose of a different commercial vaccine, we found that the mixed vaccine produced higher levels of antibodies. Our results support the conclusion that heterologous vaccination is an immune potentiator and are in agreement with previous reports in academic populations, which also corroborates the effectiveness of heterologous boosting against COVID-19 [68]. Nevertheless, the underlying mechanism for increased immunity when combining COVID-19 vaccines has not been properly established.

In addition to the heterologous vaccination, a prior infection is also known as an immune potentiator. We found higher SARS-CoV-2 IgG antibodies in the group of individuals fully vaccinated with a prior infection than in the group of individuals fully vaccinated with no prior infection. Our results corroborated other studies reporting higher SARS-CoV-2 IgG antibody levels in vaccinated individuals after a prior infection [56,69,70].

Some other host factors besides the ones considered in this study, such as the body mass index, microbiome, and comorbidities such as immunosuppression, cancer, or diabetes, were associated with alterations in antibody kinetics after vaccination or infection by SARS-CoV-2 [46,48,55]. Research on SARS-CoV-2 vaccines should prioritize identifying these immunological response variations, which might help us better understand how these factors affect COVID-19 outcomes.

## 5. Conclusions

Vaccination effectiveness and group immunity assessment are crucial to managing the pandemic, particularly in academic environments with high individual mobility.

Here, data from 529 volunteers from the IPL population were retrospectively analyzed. All samples analyzed by ELISA were found to be positive, and SARS-CoV-2 IgG antibody levels ranged from 1.2 ± 3.2 to 16.1 ± 3.2 index.

Our study demonstrated that, as expected, variables such as age, gender, type of vaccination program, and cross-vaccination were relevant for IgG antibody levels and that the boost dosage, as well as crossed vaccination, affected SARS-CoV-2 IgG levels. Further analysis of potential causes of individual variability in immunological responses and memory cell effectiveness will be crucial to understanding the influence of these variables on COVID-19 outcomes in order to define groups at risk and redirect infection control strategies.

To inform future public health initiatives, including vaccination recommendations and COVID-19 vaccination certificate lengths, continuous monitoring of the population-level IgG response following vaccination is still crucial; nevertheless, our data clearly suggest that complete vaccination programs with crossed vaccination (mRNA and viral vaccines) and boost dosage results in higher SARS-CoV-2 IgG antibody levels.

## Figures and Tables

**Table 1:** SARS-CoV-2 IgG index levels characterization by Participants’ demographic characteristics and vaccination scheme.

	Fully Vaccinated		Fully Vaccinated + Boosted (Same Vaccine)		Fully Vaccinated + Boosted (Mixed Vaccine)		Fully Vaccinated + Prior COVID-19 Infection		One Dose + Prior COVID-19 Infection	
	n (%)	IgG Mean Index (±SD)	n (%)	IgG Mean Index (±SD)	n (%)	IgG Mean Index (±SD)	n (%)	IgG Mean Index (±SD)	n (%)	IgG Mean Index (±SD)
Total										
	350 (66.2%)	7.8 (±3.0)	81 (15.3%)	9.9 (±2.7)	33 (6.2%)	11.1 (±3.1)	31 (5.9%)	10.7 (±2.5)	34 (6.4%)	9.2 (±2.6)
Sex										
Female	248 (70.9%)	8.3 (±2.8)	52 (64.2%)	10.2 (±2.7)	16 (48.5%)	10.3 (±3.0)	19 (61.3%)	10.7 (±2.6)	24 (70.6%)	9.1 (±2.8)
Male	102 (29.1%)	6.5 (±3.2)	29 (35.8%)	9.4 (±2.7)	17 (51.5%)	11.9 (±3.0)	12 (38.7%)	10.6 (±2.4)	10 (29.4%)	9.4 (±2.3)
Age (years)										
17–32	173 (49.4%)	8.4 (±3.2)	11 (13.6%)	9.4 (±2.0)	2 (6.0%)	9.5 (±0.4)	7 (22.6%)	10.2 (±2.2)	21 (61.8%)	9.4 (±2.5)
33–47	106 (30.3%)	7.2 (±2.6)	17 (21.0%)	9.4 (±2.3)	1 (3.0%)	9.8	10 (32.3%)	9.4 (±2.2)	7 (20.6%)	10.0 (±2.6)
48–62	71 (2.0%)	7.1 (±3.0)	38 (46.9%)	10.1 (±2.9)	20 (60.6%)	11.1 (±3.5)	14 (45.2%)	11.8 (±2.4)	6 (17.6%)	7.3 (±2.9)
63–7			15 (18.5%)	10.5 (±3.2)	10 (30.3%)	11.5 (±2.6)				

## Data Availability

The datasets used and/or analyzed during the current study are available from the corresponding author upon reasonable request.
